# Hydroxypyridinone-Based Metal Chelators towards Ecotoxicity: Remediation and Biological Mechanisms

**DOI:** 10.3390/molecules27061966

**Published:** 2022-03-18

**Authors:** M. Amélia Santos, Anna Irto, Péter Buglyó, Sílvia Chaves

**Affiliations:** 1 Centro de Química Estrutural and Departamento de Engenharia Química, Institute of Molecular Sciences, Instituto Superior Técnico, Universidade de Lisboa, Av. Rovisco Pais 1, 1049-001 Lisboa, Portugal; 2 Dipartimento di Scienze Chimiche, Biologiche, Farmaceutiche ed Ambientali, Università di Messina, Viale F. Stagno d’Alcontres, 31, I-98166 Messina, Italy; airto@unime.it; 3 Department of Inorganic and Analytical Chemistry, University of Debrecen, Egyetem tér 1, H-4032 Debrecen, Hungary; buglyo@science.unideb.hu

**Keywords:** hydroxypyridinones, chelating agents, metal chelators, ecotoxicity remediation, luminescent probes

## Abstract

Hydroxypyridinones (HPs) are recognized as excellent chemical tools for engineering a diversity of metal chelating agents, with high affinity for *hard* metal ions, exhibiting a broad range of activities and applications, namely in medical, biological and environmental contexts. They are easily made and functionalizable towards the tuning of their pharmacokinetic properties or the improving of their metal complex thermodynamic stabilities. In this review, an analysis of the recently published works on hydroxypyridinone-based ligands, that have been mostly addressed for environmental applications, namely for remediation of *hard* metal ion ecotoxicity in living beings and other biological matrices is carried out. In particular, herein the most recent developments in the design of new chelating systems, from bidentate mono-HP to polydentate multi-HP derivatives, with a structural diversity of soluble or solid-supported backbones are outlined. Along with the ligand design, an analysis of the relationship between their structures and activities is presented and discussed, namely associated with the metal affinity and the thermodynamic stability of the corresponding metal complexes.

## 1. Introduction

Hydroxypyridinones (HPs) are an important family of heterocyclic metal chelators, that have extensively attracted the scientific community since about five decades ago for chelation therapy, in response to human contamination by plutonium and other actinides [1]. However, a major impact on the development of HP ligands for biological applications occurred about two decades ago, namely after the disclosure and approval of deferiprone (DFP, 3-hydroxy-1,2-dimethyl-4-hydroxypyridinone), an orally active chelating drug for decorporation of toxic excess of iron(III), overcoming the disadvantage of desferrioxamine, otherwise known as deferoxamine or desferal (DFO), an orally inactive chelating drug, used for iron overload from the 1970s. Meanwhile, following new steady approaches, a large family of HP (also called HOPO) derivatives have been developed and studied, being considered as “privileged” chelating structures for many pharmaceutical applications, from therapy to diagnosis, as illustrated in several excellent review papers recently published [2,3,4,5,6].

**Figure 1:**
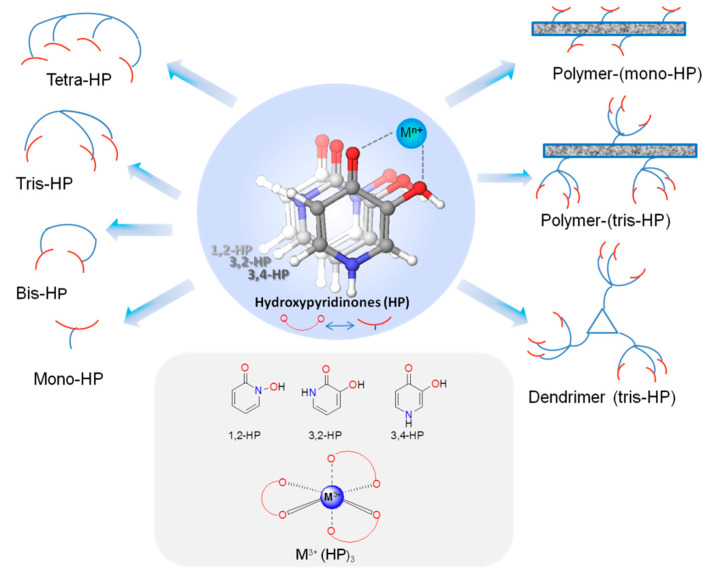
Schematic illustration of the structures of hydroxypyridinone (HP)-based metal chelators (3,4-HP, 3,2-HP, 1,2-HP), from their mono-bidentate (mono-HP) to building blocks of multi-HP ligands, and also typical HP-metal (M^3+^) coordination mode.

This family of bidentate chelators is characterized by an aromatoid 6-member *N*-heterocycle with two exocyclic *O*,*O*-donor atoms, provided by *ortho*-positioned keto and hydroxy groups, which endow them with capacity for the formation of very stable complexes with *hard* metal ions, such as iron(III). Inside this family of HP-based chelators, there are three classes of positional isomers, namely, the 1-hydroxy-2-pyridinones (1,2-HP), the 3-hydroxy-2-pyridinones (3,2-HP) and the 3-hydroxy-4-pyridinones (3,4-HP) (Figure 1), as well as the related *O*-heterocyclic 3-hydroxy-4-pyrones. The 1,2-HPs were the first discovered class of HPs, specially intended to the specific sequestration of radioactive actinides [1]. Their development strategy followed a siderophore mimetic approach, associated with the analogy of 1,2-HP with the hydroxamate chelating moieties of the naturally occurring siderophore desferrioxamine, and also on the similarities between Fe(III) and Pu(IV). Later on, also based on the same biomimetic approach towards new stronger Fe(III) sequestering agents, a steady progress resulted in the subsequent discovery of 3,2-HP and afterwards 3,4-HP, which structures can be considered between a cyclic hydroxamate and a *N*-heterocyclic analogue of catechol, the bis(*ortho*-phenolate)-chelating moiety of the siderophore enterobactin [1]. These three classes of HPs differ in the placement of the (*O*,*O*)-donor group around the central pyridinic ring. The increased distance between the (*O*,*O*)-donor group and the pyridinic nitrogen atom (1,2-HP < 3,2-HP < 3,4-HP) is reflected by the parallel basicity increase in the corresponding hydroxy groups and in electronic density at the coordination atoms and concomitant increase in affinity for *hard* metal ions, namely at neutral pH conditions [2,3]. This broad family of chelators can easily be prepared and functionalized, according to the envisaged multiple purposes, and so these metal chelating motifs have been considered as very promising building block structures for the rational engineering of a panoply of metal sequestering agents of biological relevance, namely for medicinal and environmental applications [1,2,3,4,5]. The higher metal chelating affinity of 3,4-HPs under neutral pH conditions has been the main rational for their major recent HP-based developments associated with potential medicinal applications, either as chelators (e.g., for chelatotherapy in *hard* metal overload or antimicrobial activity) or as metal complexes/metallodrugs (e.g., for imaging diagnostic probes, as Gd and ^68^Ga complexes [6], or for anti-cancer therapy, as Ru and Os complexes) [2,4]. However, a lot of efforts have also been recently focused on environmental-related applications of HPs, namely for remediation of metal ecotoxicity of living beings and other different biological matrices or even for the detection and removal of anions from contaminated waters. Among these, the decorporation of excess-specific toxic *hard* metal ions, such as aluminum(III) and plutonium(IV), as well as the downstream decontamination of biological matrices, is of paramount importance. In fact, aluminum can accumulate in the body due to diverse environmental insults, and its toxicity is associated with bone disorder and neurological diseases. Additionally, plutonium and other radioactive actinides, from nuclear waste and fission products, can accumulate in aqueous or atmospheric environments and enter the body, resulting in cancer or genetic deformations.

Therefore, taking into account that the major amount of recently published work in papers and reviews is related to the potential applications of HPs in the medicinal chemical area, herein major attention is focused on the revision and analysis of the most recent worldwide publications (last 5–6 years) mainly associated with the use of this family of compounds towards the remediation of ecotoxicity caused by *hard* metal ions. Major care is given to ligand design and the thermodynamic stability of the corresponding metal complexes, considering the specificity of the chased metal ions and the diverse applications. The main sets of HP ligands are schematically presented in Figure 1, starting from the mono-hydroxypyridinones (bidentate chelators with only one HP chelating moiety, e.g., DFP drug) to the bis-, tris-, tetra- and poly-hydroxypyridinones, which include, respectively, 2, 3, 4 and multiple chelating units, linked to soluble backbones and to dendrimeric or solid polymeric systems. The charge of the chased metal ion determines the optimum number of chelating units necessary for the metal wrapping and full coordination, in order to guarantee the maximum sequestration capacity and thermodynamic/kinetic stabilities of the metal complexes. However, the number of chelating moieties may also be dependent on the envisaged applications. In fact, a high number of HP units in a molecular entity may induce limitations on the pharmacokinetic descriptors that may hamper their bioavailability or the targeting of specific organs. Thus, in metal decorporation applications, mono-hydroxypyridinone-based chelators (as DFP) may be used, although requiring higher dosage concentration to guarantee full metal coordination and thermodynamic stability of the metal complex inside the body. However, for detoxication of aqueous fluids or sensing applications, the use of larger size polydentate molecules has the advantage of increasing the metal sequestration and overcoming the disadvantage of potential ligand excess toxicity. This can be accomplished by either using a discrete number (2–4) or a multiple number of HP chelating units attached to an anchoring skeleton or to polymeric systems as solid matrices. Along the text, comparisons are made in terms of structure activity relationships, namely aimed to correlate structural variations, as the number and type of substituent groups and HP units (in some cases a cocktail of different chelating units is used) as well as the anchoring backbone topologies, with the thermodynamic stability of the corresponding metal complexes and the metal detoxication effects.

## 2. Mono- and Bis-(3-hydroxy-4-pyridinone) Ligands

### 2.1. Mono-Hydroxypyridinones

**Figure 2:**
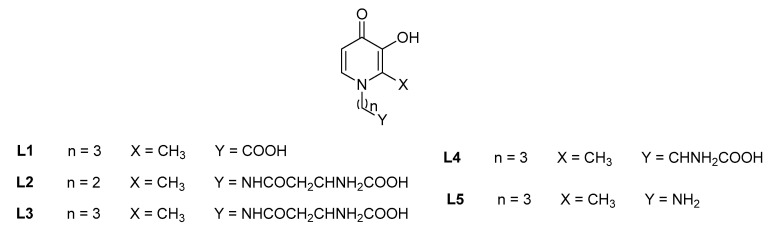
Mono-hydroxypyridinone ligands employed for thermodynamic studies on metal chelation.

**Figure 3:**
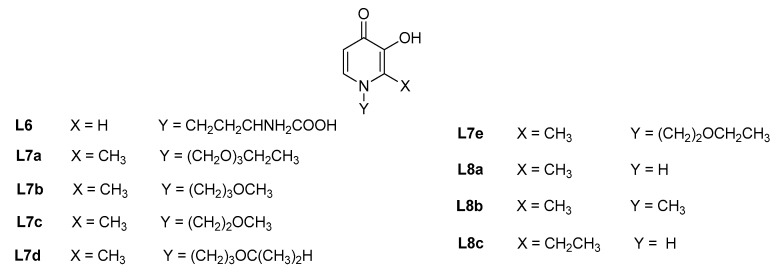
Mono-hydroxypyridinones used for Fe^3+^ determination in natural matrices and as potential new fertilizers.

**Figure 4:**
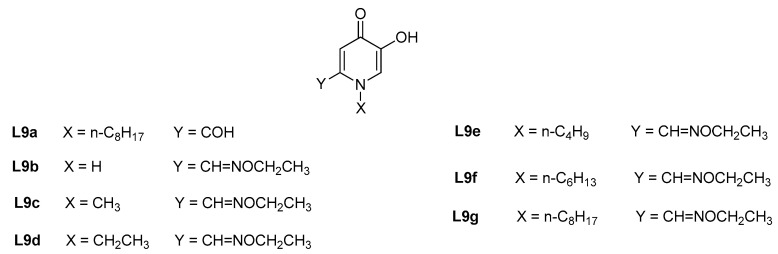
Mono-hydroxypyridinone ligands employed as tyrosinase inhibitors as anti-browning agents in fruits and vegetables.

In the last six years, many 3,4-HP mono-hydroxypyridinone-based ligands (mono-HPs, Figures 2–4), enclosing various extrafunctional groups were developed envisaging potential different environmental applications as for the remediation of eco-toxicity of specific *hard* metal ions, the formulation of new fertilizers and also the development of tyrosinase inhibitors used as anti-browning agents in fruits and vegetables.

#### 2.1.1. Thermodynamic Studies on Metal Chelation

Bifunctional 3-hydroxy-4-pyridinones (**L1**–**L5**, Figure 2), featured by a single 3,4-HP chelating core and various functional groups attached to the pyridinone moiety by different *N*-alkyl spacers, were synthesized and thermodynamically evaluated as possible strong chelators of *hard* metal cations of environmental relevance (Al^3+^, Fe^3+^) [7,8], using potentiometric and spectrophotometric techniques; the potential thermodynamic competition, and eventually depletion, of other *hard* (Ca^2+^, Mg^2+^) and *borderline* (Zn^2+^, Cu^2+^) character metals of environmental interest was also evaluated [8,9,10]. The main experimental conditions chosen by the authors was *I* = 0.15 M in NaCl_(aq)_ and *T* = 298.15 K. Sodium chloride was selected as ionic medium, since it is the main inorganic constituent of many natural fluids [11,12], or it can also be found in soils as contaminant (saline soils) [13]. In some cases, studies at different ionic strengths (*I* = 0.50–1.00 M) and temperatures (*T* = 288.15–310.15 K) were reported, providing useful information for the determination of thermodynamic parameters allowing us to simulate the real conditions of natural fluids and other environmental matrices [8,9,10].

Comparisons between the different M^n+^/mono-3,4-HP systems [7,8,9,10], and also with the literature data reported for deferiprone (3-hydroxy-1,2-dimethyl-pyridin-4(1*H*)-one, dmmp, DFP, **L8b**, Figure 3) [14], widely diffused as *hard* metals chelating agent, were performed based on the pM parameter (pM = −log [M^n+^], for *c*_Metal_ = 1 μM and *c*_Ligand_ = 10 μM) [15], to gain information on the ligands affinity towards the different metal cations, as reported in Table 1. In the literature, the pL_0.5_ parameter [16], indicating the total concentration of ligand required to sequester the 50% of M^n+^ if present in trace (10^−6^ μM) in the solution, was proposed to compare the sequestering ability of 3-hydroxy-4-pyridinones towards one or more metal ions. The pL_0.5_ calculation can find applications for the remediation of polluted systems, in processes of water treatment and in the activities involving the employment of a chelating agent, since the knowledge of the ligand concentration to be used can be useful for optimization of the working conditions [8].

The five mentioned bifunctional mono-(3-hydroxy-4-pyridinones) (**L1**–**L5**, Figure 2), were developed and tested as chelators of several metal ions (Al^3+^ [7], Fe^3+^, Cu^2+^ [8], Zn^2+^ [10], Ca^2+^, Mg^2+^ [9]) and the sequestering ligands are featured by carboxylic (**L1**), amide-amino-carboxylic (**L2**, **L3**), amino-carboxylic (**L4**) and amino (**L5**) groups in the *N*-alkyl chain attached to the pyridinone ring. **L1** was proposed as a good aluminum(III) chelating (Table 1) and sequestering agent (pL_0.5_ = 6.3 (Al^3+^), 4.8 (Cu^2+^), 2.5 (Zn^2+^), <1.0 (Ca^2+^)), excluding the possibility of an effective ligand M^2+^ chelation instead of the Al^3+^ remediation. The amide-amino-carboxylic **L2** ligand was developed as a strong Fe^3+^ chelator, with an iron(III) affinity about ten orders of magnitude higher than with aluminum(III), in turn stronger than with divalent metal cations and a better Fe^3+^ and Zn^2+^ chelating agent than DFP (pFe = 19.3, pZn = 6.2) [14]. **L3**, with an extra -CH_2_ group in the *N*-alkyl chain with respect to **L2**, and **L4**, a 3,4-HP derivative of *L*-ornithine, exhibited quite a good aluminum(III) chelating capacity, between two and five logarithmic units stronger than Cu^2+^ and Zn^2+^, Ca^2+^, Mg^2+^, respectively. The amino mono-3-hydroxy-4-pyridinone **L5** was proposed as a much stronger Fe^3+^ chelator (Table 1) and good sequestering agent (pL_0.5_ = 7.6 (Fe^3+^), 5.4 (Al^3+^), 4.6 (Cu^2+^), 2.0 (Zn^2+^), <1.0 (Ca^2+^, Mg^2+^)) with respect to Al^3+^ and M^2+^, excluding the possibility of a preferential chelation of aluminum(III) and/or divalent metal cations ligand instead of that with iron(III).

#### 2.1.2. Determination of Iron(III) in Environmental Matrices

Iron is an essential element for living organisms but, when it is present under excess concentration in natural waters, aesthetic effects such as odor, color and taste change [17], as well as structural problems such as damage of water pipelines can occur [18]. Therefore, the Fe^3+^ determination and quantification in these matrices is extremely important to evaluate the metal distribution in the environment. The recommended methods used for this purpose are usually based on spectrophotometry [19], atomic absorption or inductively coupled plasma spectrometry [20,21], chemiluminescence [22] and colorimetric [23] procedures. In many cases, various drawbacks connected to the employment of the mentioned techniques or the use of toxic reagents [18,24], led the scientific community to the development of new methods based on a green chemistry perspective [18].

A simple and crude aqueous extract from leaves of *Leucaena leucocephala* (Lam.) de Wit, a lead tree abundant in tropical areas containing a hydroxypyridinone, namely mimosine, (**L6**, Figure 3) was employed as natural color reagent for the Fe^3+^ assay, obtaining very similar calibration curves, limits of detection (LOD, Table 2) and of quantification (LOQ, Table 2), with respect to the commercial mimosine [25]. The extract method was also successfully tested in real samples of blood tonic obtaining data in good accordance with those already reported in literature using the atomic absorption techniques and other traditional or synthetic chelators [26], with a statistical t_observed_ value of 0.008, much lower than the t_critical_ = 2.78 at 95% of confidence level. This new method for the iron(III) determination could be employed in remote areas, where the synthesis of Fe^3+^ chelators is not accessible, otherwise offering an alternative, cheap and green chemical method for the metal quantification [25].

On the other hand, in the last decade, the design of *N*-alkyl-3-hydroxy-4-pyridinones has provided a “first-generation” of 3,4-HP to be employed as chromogenic reagents for iron(III) determination [30,31], but the intermediate or low solubility of the compounds in water media, together with the high detection limits and low sensitivity of the methods used for the metal examination, have challenged the researchers to develop a new “second generation” of 3-hydroxy-4-pyridinones, that could assure a higher ligand solubility in water than the “first-generation” products, and provide analytical methods for Fe^3+^ determination with lower detection limits and higher sensitivity.

The 3-hydroxy-4-pyridinone attached to a polyethylene glycol chain, MRB12 (**L7a**, Figure 2), previously synthesized and tested for the iron(III) determination in natural waters using a sequential injection method [30], was recently employed for the metal quantification in real river waters, developing a method based on a selective microsequential injection lab-on-valve (µSI-LOV) system [18]. This method was validated by using certified reference waters and both the characteristic validation parameters (LOD, LOQ, Table 2), were much lower than the maximum Fe^3+^ concentration (*c*_Fe3+_ = 5.37 µM) recommended by the Environmental Protection Agency (EPA) for drinking waters [17]. An improvement in the sample volume (150 µL) and the ligand concentration (0.88 mM) was also obtained with respect to the previous sequential injection method [30]. Four “second generation” of hydrophilic 3,4-HPs, containing ether groups, called MRB13, MRB14, MRB15 and MRB16 (**L7b**–**L7e**, Figure 3), were synthesized using an innovative and sustainable synthetic method (employment of microwave heating instead of oil-bath reflux) [27]; their use in the sequential injection method for the selective Fe^3+^ investigation in natural waters, resulted in LOD (Table 2) and sensitivity values (13.0-13.8 mM) quite similar to previously obtained data by the same research group for MRB12 chelator with the same analytical techniques. These results suggest that the structural differences introduced, with respect to MRB12, do not compromise the chromogenic chelator performances for Fe^3+^ determination in natural waters. A disposable microfluidic paper-based analytical device (μPAD) [28] was developed employing the five mentioned chelators as color reagents, owing to their lower toxicity with respect to 1,10-phenantroline (the colorimetric reagent typically employed for these types of applications [24]) and high water solubility, which increased the 3,4-HPs applicability as μPAD reagents. This method for the metal detection in river and tap water, not involving a sample pre-treatment, resulted to be effective, in situ, portable, disposable, accurate, easily operated and cheap, in accordance with LOD and LOQ World Health Organization guidelines [28], also showing a consumption of 3,4-HPs lower than 0.2 mg per analysis. A selective flow-based method for the Fe^3+^ spectrophotometric quantification in fresh and marine waters with variable salinity content was developed using the MRB13 chelator [29] and two working strategies, including and not employing an on-line solid-phase extraction (SPE). Water samples with high salinity and/or a low metal concentration were investigated by means of the first approach using a NTA resin column incorporated in the system, for the sample matrix clean-up and/or Fe^3+^ pre-concentration; the second strategy was employed for waters with opposite properties. Based on LOD values (Table 2) determined with the two working strategies, a higher efficiency of the on-line SPE method, with a low reagent consumption and low effluent production, was observed. The possibility of system portability makes this method appropriate for in situ Fe^3+^ monitoring in aqueous media.

#### 2.1.3. Formulation of New Fertilizers

Fe^3+^ in plants plays fundamental roles participating in the photosynthesis, in the chlorophyll synthesis, in the nitrogen fixation and in enzymes activation processes. A deficiency of this metal could interfere and hinder these activities and cause the Iron Deficiency Chlorosis (IDC), leading to the yellowing of leaves, the decrease in their area and in the shoot and root dry weight, a slow plant growth and a high oxidative stress [32]. Soybean (*Glycine L. max*) plants, cultivated in calcareous alkaline soils, are particularly susceptible to this pathology and use a reduction-based strategy for increasing the metal uptake. The application of Fe^3+^ fertilizers based on the use of chelating agents such as polyaminocarboxylic acids, such as EDTA and EDDHA, is the most common method employed for the treatment of IDC, but several side effects led the scientific community to look for new possible iron(III) fertilizers. The 3-hydroxy-4-pyridinones can be considered good alternative chelators for the formulation of Fe^3+^ fertilizers, as already observed in 2016 for the Fe(mpp)_3_ complex (mpp, **L8a**, Figure 3) which showed a stronger efficacy on IDC prevention in soybean plants when compared with the commercial Fe(EDDHA) product [33]. In the last couple of years, various 3-hydroxy-4-pyridinone chelating agents were developed, using literature synthetic procedures, and tested as possible fertilizers in soybean [32,34]. Three Fe(3,4-HP)_3_ chelates, Fe(mpp)_3,_ Fe(dmpp)_3_ (dmpp, **L8b**) and Fe(etpp)_3_ (etpp, **L8c**, Figure 3), different from each other in the substituents and functional groups were developed and tested for IDC prevention. The metal complexes resulted in significant Fe^3+^ delivery in plants with an efficacy following the trend: **L8a** > **L8b** > **L8c**, underlining that little differences in the ligand structures are important to discriminate the best chelating agent to prevent and/or treat IDC. Fe(mpp)_3_ exhibited the greatest morphological, physiological and gene expression performances; a significant efficiency of the complex at *c*_Fe(mpp)3_ = 10 μM was observed at variable pH of hydroponic culture medium, half dose with respect to that described by the same research group in 2016 [33], lower than the commercially available fertilizers. In alkaline soil conditions, mpp demonstrated a good efficacy to provide iron(III) to plants, to favor the metal reduction and improve its uptake and translocation from the root to the leaves. The foliar application of Fe(mpp)_3_ chelate in soybean plants, grown in hydroponic conditions from seed to maturity, produced a more satisfactory effect, a higher versatility, lower environmental costs and lower dosage requirements than those necessary employing Fe(EDDHA), as well as an increase in chlorophyll content, root biomass, trifoliate metal concentration, in leaf Ferritin gene expression. The new metal chelate favored a higher resistance to Fe^3+^ deficiency in root and in shoot and, at complete maturity, produced a significant rise in seed yield and an increased accumulation of essential metals for the plants growth than commercial products [34].

#### 2.1.4. Tyrosinase Inhibitors as Anti-Browning Agent in Fruits and Vegetables

Several foods can readily turn brown through processing and storage, causing a worsening in their quality and commercial value decreasing their shelf life. Therefore, the prevention of this problem results in a very relevant issue to be solved by the scientific community. In fruits and vegetables, the undesirable browning process is caused by tyrosinase [35], a metalloenzyme featured with two copper cations (Cu^2+^) in the active site [36]. It is known as a rate-limiting enzyme in the biosynthesis of melanin pigment [37]. It catalyzes the hydroxylation of monophenols to *o*-diphenols, as well as their subsequent oxidation to *o*-quinones [38], which, in turn, can spontaneously polymerize and form melanin [39]. Similarly, food components such as amines, amino acids, peptides and proteins, can damage the essential amino acids, reducing the fruits and vegetables digestibility with a loss in the activity of proteolytic and glycolytic enzymes leading to damage in the food’s nutritional quality [40,41]. Therefore, tyrosinase inhibitors were developed to prevent or delay the browning of fruits and vegetables, but many of these molecules did not find concrete application due to drawbacks related to significant toxicity, as well as low activity and stability. Kojic acid (KA), a naturally occurring 3-hydroxy-4-pyrone ligand, is a tyrosinase inhibitor, that has been used as an anti-browning agent in food that quickly changes color [41], exhibiting an efficient Cu^2+^ chelation and reduction in Cu^+^ at the enzyme active site [42]. 3-Hydroxy-4-pyridinones (3,4-HP) have been considered as possible candidates for tyrosinase inhibition, since they are structurally similar to KA and are able to interact with Cu^2+^ with a higher stability and metal affinity with respect to KA. Actually, literature values of log *K*_CuKA_ = 6.60 and pCu = 7.3 (50% dioxane medium, *T* = 298.15 K) were reported [43], while for DFP (**L8b**, Figure 3), log *K*_CuDFP_= 10.42 and pCu = 10.7 were determined in aqueous medium at the same temperature [44].

In this context, new hydroxypyridinone derivatives, synthesized from kojic acid (KA) and featured by a formyl group (**L9a**, Figure 4) or oxime ether moieties and different alkyl chain bound to the *N*-heterocyclic ring (**L9b**–**L9g**, Figure 4) [41,42], were developed and checked as inhibitors of mushroom tyrosinase (mono- and di-phenolase activity) as well as anti-browning products in potatoes and fresh-cut apples, respectively. The results can be compared with those already published in literature for KA by different authors (IC_50 monophenolase_ = 12.24 μM [42], 15.59 μM [42]). **L9a** exhibited a stronger mushroom tyrosinase inhibition on the mono- and diphenolase activities (IC_50 monophenolase_ = 1.33 μM, IC_50 diphenolase_ = 7.83 μM), a higher stability (log *K*_Cu(L9a)_ = 12.70) and copper affinity (pCu = 17.15) when compared with KA. This compound is also able to hamper the potatoes browning by decreasing the formation of *o*-quinone [38]. Among the oxime ether HPs (**L9a**–**L9g**), **L9f** and **L9g** exhibited the strongest inhibitory effect on mushroom tyrosinase (IC_50 monophenolase_ = 1.60–2.04 μM, IC_50 diphenolase_ = 7.99–13.89 μM), thus higher than KA. Compound **L9g** was also able to prolong the shelf-life of slices of fresh-cut apple by controlling and retarding the browning effect.

### 2.2. Bis-Hydroxypyridinones

**Figure 5:**
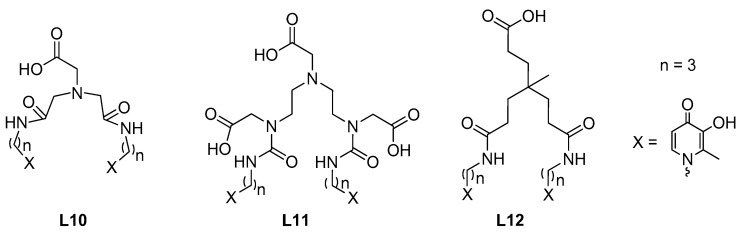
Bis-3-hydroxy-4-pyridinones employed for possible environmental applications.

To further increase the efficacy of mono-hydroxypyridinones, as metal sequestering agents, in the last six years, three bis-3-hydroxy-4-pyridinones (bis-3,4-HPs) were developed as potential selective chelators of divalent and trivalent metal cations of environmental relevance, for possible sensing or targeting purposes. These polypodal ligands are featured by polyaminocarboxylic acids (NTA(PrHP)_2_, DTPA(PrHP)_2_, named **L10** and **L11** in Figure 5) [45,46], or a triscarboxylic acid (KC21, **L12** in Figure 5) [47], as central cores (anchors) attaching two 3-hydroxy-4-pyridinone arms.

The reported thermodynamic studies on the mentioned bis-3-hydroxy-4-pyridinones (L) and the speciation models revealed the formation of stable mononuclear and binuclear protonated species (M_p_L_q_H_r_; p, q = 1–2, r = 1–4) with 1:1 and 1:2 stoichiometry (ML, ML_2_), as well as mixed hydroxo (MLOH, M_2_L_2_(OH)_2_) species at the highest pH ranges. The presence of polynuclear complex species was detected in the case of **L10** and **L11**. Since these complexes should not be formed at the trace concentration in which the pL_0.5_ is calculated, this parameter could not be used for comparisons between the different metal-ligand systems but only the pM values (Table 3).

At micromolar conditions (*c*_Ligand_ = 10 μM, *c*_Ligand_/*c*_Metal_ = 10), **L10** and **L11** exhibited a stronger chelating capacity for Fe^3+^ and Al^3+^, and a lower affinity for Zn^2+^, Cu^2+^ (pCu = 19.4, 17.3) and Ca^2+^ (pCa = 17.2, 14.6). In the case of **L12**, Zn^2+^ was the only divalent metal cation investigated and the metal efficacy followed the trend: Fe^3+^ > Al^3+^ >> Zn^2+^, excluding a Zn^2+^/M^3+^ thermodynamic competition. **L10**, **L12** and in some cases, also **L11**, turned out to be better Fe^3+^ and Al^3+^ chelators than DFP [14], desferrioxamine (DFO, mainly towards Al^3+^) [48], transferrin [49,50] and polyaminocarboxylic acids such as NTA, EDDA, DTPA and DOTA [51], widely employed as iron(III) and aluminum(III) chelators. Furthermore, **L10** and **L11** proved to be stronger Zn^2+^ chelating agents than DFP and DFO, as well as EDDA and DTPA. **L10** also exhibited a higher Fe^3+^, Al^3+^ and, when investigated, Zn^2+^ chelating capacity with respect to other bis-3,4-HPs previously developed, such as IDAPr(3,4-HP)_2_ [52], EDTAPr(3,4-HP)_2_ (mainly towards Al^3+^, Zn^2+^) [53], IDAPipPr(3,4-HP)_2_ [54]. Furthermore, **L10**, **L11** and **L12** showed a better Al^3+^ affinity with respect to a combined chelation system featured by a bis- and a mono-3,4-HP, namely IDAPipPr(3,4-HP)_2_ and **L4** [54].

## 3. Poly-Hydroxypyridinones

Several *hard* metal ions are known to contaminate different water resources as well as soils and sediments. In order to increase the metal chelation capacity of simple bidentate chelators, containing two high electronic density oxygen donor atoms, as the mono-hydroxypyridinone ligands, a considerable amount of research has been dedicated to the development and study of polydentate chelators, namely tetradentate (bis-hydroxypyridinones, above described in Section 2.2), hexadentate (tris-hydroxypyridinones) and octadentate (tetra-hydroxypyridinones), described in this section. These compounds are obtained by appending two to four bidentate chelating moieties to selected backbone skeletons with different topologies (e.g., linear, tripodal, tetrapodal, cyclic, macrocyclic), leading to improvements in the thermodynamic and kinetic stability of the metal complexes and to the elimination of dilution effects with beneficial decrease in dosage need.

Recognition of anions in polluted water resources is also another important topic concerning the development of probes able to detect, sequestrate and transport a variety of anions present in polluted water streams. In recent years, different receptors have been proposed, by matching the hardness of the targeted anionic base to that of the corresponding metal receptor and guaranteeing that the coordination core has one or more open coordination sites in order that the anion can coordinate to the metal center. To obtain anion selective receptors, factors such as ligand geometry, the type of chelating moieties, steric hindrance at the binding site and Coulombic interactions were found to influence the performance of the compounds.

### 3.1. Tris-Hydroxypyridinones

In the last five years, hexadentate hydroxypyridinones, along with some hydroxypyrone derivatives, have been developed as strong metal chelators with potential application in metal remediation, as analytical reagents in spectrophotometric/colorimetric or luminescent detection of contaminants or for anion recognition and removal from polluted surface, ground and coastal waters eventually associated with eutrophication processes.

#### 3.1.1. Metal Chelators

**Figure 6:**
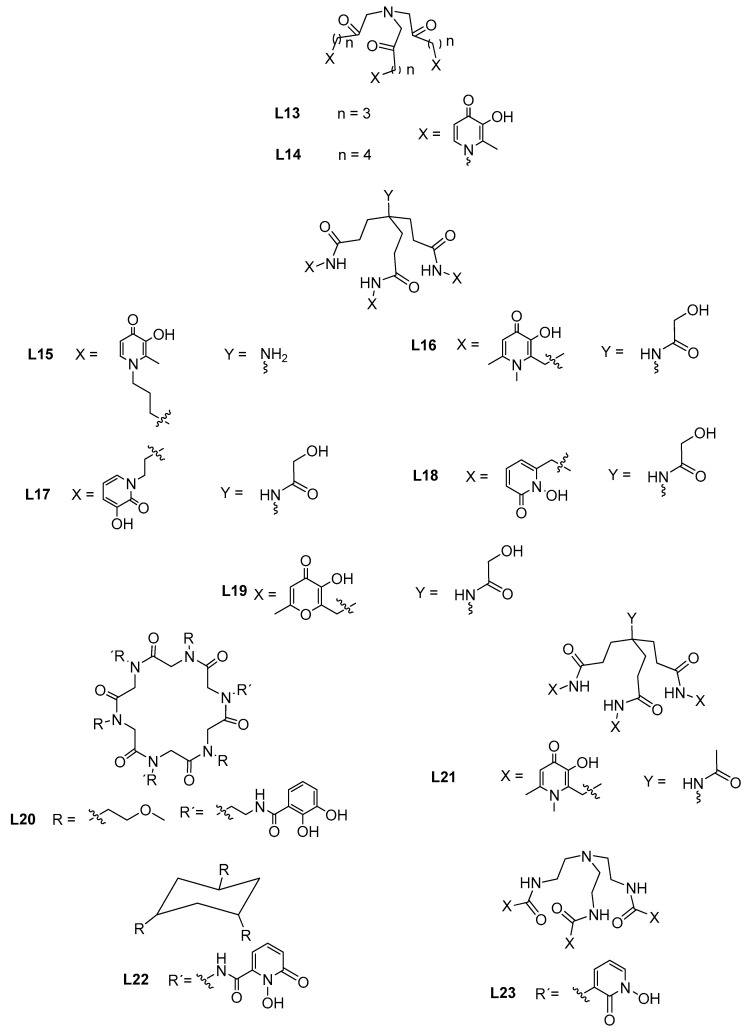
Molecular structure of selected hexadentate compounds as metal chelators.

Researchers have long been interested in developing effective hydroxypyridinone-based iron chelators due to the importance of this essential metal ion in living organisms, playing vital roles in redox processes such as nitrogen fixation, oxygenation, respiration and photosynthesis. On the other hand, though aluminum is a non-essential element it is released in the environment by different anthropogenic activities associated with industrial, alimentary or pharmaceutical sources. Although most of the developed compounds have been initially proposed for chelation therapy purposes, they may also have eventual application in the removal of metal contamination from diverse water resources. A hexadentate 3,4-HP tripodal compound (NTA(PrHP)_3_, **L13**, see Figure 6) was recently developed and, although with high iron and aluminum chelation abilities (pFe = 26.3, pAl = 19.8), meaning 7 and 4 orders of magnitude higher than DFP [55], it rendered slightly weaker than another analogous hexadentate compound NTA(BuHP)_3_ (**L14**) (pFe = 27.9, pAl = 22.0) [56], attributed to some linker truncation and accordingly more strained metal coordination geometry. Another hexadentate tris-3,4-HP metal chelator (KC18, **L15**) was quite recently reported, with much stronger iron and aluminum chelating capacity (pFe = 29.5, pAl = 23.8) than NTA(PrHP)_3_ (**L13a**) due to the less distorted coordination core [47]. Furthermore, this ligand can be extra-functionalized through the backbone amino group to provide capacity for biotargeting or for grafting on solid matrix and nanomaterials, thus expanding its potential clinical or environmental applications.

The conjugation of three 3,4-HP, 3,2-HP, 1,2-HP or 3-hydroxy-4-pyranone units with the same extra-functionalizable tripodal scaffold, was also recently reported for four compounds (**L16**–**L19**, see Figure 6). The corresponding pFe values follow the sequence **L16** (pFe = 27.6) > **L18** (pFe = 26.8) > **L17** (pFe = 23.9) > **L19** (pFe = 23.5), thus demonstrating the superior capacity of hexadentate 3,4-HP and 1,2-HP over the 3,2-HP analogue as iron scavengers, having higher or similar iron chelation capacity than the clinical drug desferrioxamine (DFO, pFe = 26.5) [57,58]. This pFe sequence is not in accordance with the corresponding one for simple bidentate compounds (3,4-HP > 3,2-HP > 1,2-HP > 3-hydroxy-4-pyranone), which may depend on other structural differences, but explain the interest in further exploring hexadentate tris-1,2-HP; moreover, some solubility issues may arise for the iron complexes of **L16** and **L18** at neutral pH and concentrations superior to 0.1 mM.

A cyclic hexapeptoid scaffold inspired by ferrichrome A and containing a tris-(catecholate)-binding system, was also quite recently used in the design of hexadentate compounds in order to associate good iron chelation with resistance to degradation by hydrolytic enzymes. It was found that the introduction of amide groups next to catechol moieties in R^1^ substituents, as well as the orientation of the two hydroxyl groups towards the center of the macrocycle, was important in the stabilization of the Fe^3+^-**L20** complex, through the establishment of intramolecular hydrogen bonds between amide protons and catechol oxygen atoms [59]. **L20** is able to form an iron complex more stable than Fe^3+^-DFO, but the lability of the peptoid skeleton and the low solubility in aqueous media, prevents eventual environment applications.

Two tripodal ligands with 3-hydroxy-4-pyrone chelating moieties have also been recently developed by attaching three naturally occurring kojic acid (5-hydroxy-2-(hydroxymethyl)-4*H*-pyran-4-one) units, through its 2-hydroxymethyl group to two different backbone skeletons, namely the tris(2-aminoethyl)amine (TREN) [60] and the benzene-1,3,5-tricarboxylic acid [61]. They were both studied for their thermodynamic stability with Fe(III), Al(III) and other metal ions. Both ligands are remarkably easy to make and present good chelating capacity and selectivity for iron compared to other metal ions; however, as expected, the corresponding iron complexes present lower stability than found for most of the analogous tripodal tris-(3,4-HP) derivatives. On the other hand, water solubility issues and also the lability of the ester linkers of the benzene-carboxylate derivative limit their applications as iron sequestering agents.

Iron can be introduced in the ocean by dust deposition and the levels in seawater are in the order of nM concentration, which make its quantification difficult besides the quite complex nature of the seawater matrix (diversity of elements and high salinity). So, new analytical techniques with low detection limits are required and, in that line, a micro sequential injection lab-on-valve technique with solid phase spectrophotometry (SPS) was proposed for Fe^3+^ quantification [62], using quite a powerful hexadentate tris-3,4-HP colorimetric reagent, named CP256 (**L21**, pFe = 29.8 [63]). This technique (LOD = 0.13 μM, LOQ = 0.43 μM) was successively applied to different water samples (river, ground, estuarine, tap, seawater) allowing direct determination on the transparent nitrilotriacetic acid (NTA) super flow resin by selective iron retention on the sorbent (pre-concentration) and discarding of the sample matrix, avoiding elution and washing steps as well as decreasing analysis time.

Concerning lanthanide complexation, a new flexible tripodal siderophore-mimic hexadentate chelator, bearing three 1,2-HP units attached to a cyclohexane backbone (TACH-1,2-HOPO, **L22**) [64] was recently developed and was able to chelate trivalent lanthanide ions (La^3+^, Gd^3+^, Lu^3+^). By using both thermodynamic experimental studies and molecular modeling analysis for geometry prediction of the complexes, a predominance of ML species in aqueous solution and a distorted tricapped trigonal prism geometry for the coordination core were found. **L22**, which involves non-covalent interactions such as hydrogen-bonding and π-stacking, may also have the potential to be used as a lutetium sensor.

Due to the development of clean-energy technologies based on rare earth elements (Sc, Y, La-Lu), separation techniques for these elements became an important goal. Compound **L23,** designed with an exchange of the positions of the carbonyl and hydroxyl groups in the 1,2-HP moieties (see Figure 6), was proposed as a chelator in the separation of binary mixtures of some rare earths (La, Nd, Dy) via selective precipitation from aqueous media with controlled pH value, in a single complexation/separation step [65]. Using High-Throughput Experimentation (HTE) for 1:1 mixture, separation factors (SF) of SF_Nd/Dy_ = 213 and SF_La/Nd_ = 16.2 were obtained, as well as 84% ligand recovery.

#### 3.1.2. Recognition and Removal of Anions

**Figure 7:**
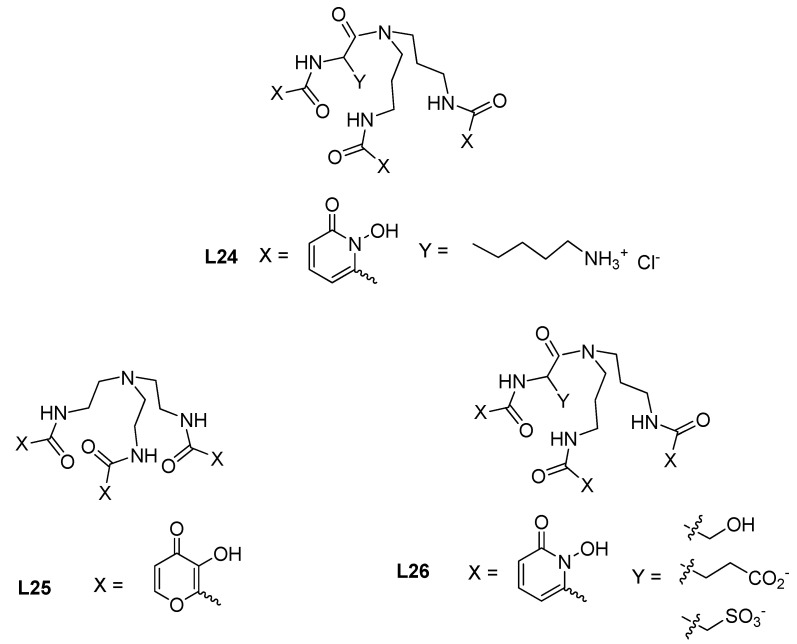
Molecular structure of selected hexadentate compounds for recognition and removal of anions from water resources.

Cyanide ion can be considered as a *soft* base according to HSAB, and it is highly toxic for humans and the environment. A luminescent probe, based on a europium complex of a hexadentate tris-1,2-HP compound (Lys-HOPO, **L24**, see Figure 7) that works in pure aqueous media, was proposed for the detection and quantification of this anion [66]. The complex Eu^3+^-**L24** acts via direct and reversible coordination of cyanide to Eu^3+^, thereby displacing three inner-sphere water molecules and increasing the luminescence intensity. The turn-on response of this probe and its selectivity towards cyanide in the presence of other relevant anions in the water column (e.g., chloride, bromide, iodide, acetate, sulphate, nitrate) make this probe a promising candidate for environmental applications. The interference of competing anions, such as fluoride, carbonate or phosphate, can be eliminated by the addition of a soluble calcium salt.

Phosphates are components of fertilizers that can accumulate in water resources due to agricultural runoffs. The eutrophication of surface and coastal waters is provoked by an excess of nutrients in water that originate algal blooms and dead zones, therefore provoking substantial environmental, economic and health problems. Since lanthanide ions are both hard and labile, they may enable efficient and quick recognition of phosphates in water and thus a Gd^3+^ complex of a hexadentate tris-maltol-based compound (TREN-MAM, **L25**, Figure 7) was reported in the literature [67]. **L25** can strongly catch inorganic phosphate directly from water at neutral pH and release it, under acidic medium (pH < 2, stable complex), for recycling of phosphate from water resources in agricultural areas. The phosphate species HPO_4_^2−^ forms a ternary complex with Gd^3+^-**L25**, replacing both inner-sphere water molecules. This gadolinium-based supramolecular receptor can be used at least 10 times in the referred pH-dependent recycle system, being a highly selective catch-and-release system for phosphate over other anions (bicarbonate, acetate, sulphate, nitrate, nitrite, bromate, fluoride, chloride and bromide). Supramolecular receptors consisting of Eu^3+^ complexes of tris-1,2-HP hexadentate compounds, containing one single-charged group (–NH_3_^+^, –CO_2_^−^, –SO_3_^−^) or a neutral hydrogen-bonding moiety (–OH) inserted in the backbone (**L24** and **L26**), were also proposed as a way of tuning the affinity for phosphate of the metal receptor. It was observed that a peripheral group positively charged or a hydrogen-bonding -OH moiety promotes binding of the phosphate to the receptor but, interestingly, these moieties, positioned peripherally to the metal’s open coordination sites, do not affect their selectivity for phosphate over competing anions [68]. Moreover, the large luminescence response of these europium complexes makes them promising agents for the detection and remediation of phosphate in complex water media. In another kind of study, tripodal gadolinium complexes, with different bidentate chelating moieties, were developed and their affinity for anions in water at neutral pH was evaluated by longitudinal relaxometry measurements in order to determine the role of the coordinating motif on the supramolecular recognition of anions [69]. For each complex, two sites on the Gd^3+^ were left open to coordination by water molecules or anions. It was found that the lower the stability of the Gd^3+^ complex, the higher its affinity for anions: TREN-1,2-HOPO forms a very stable Gd^3+^ complex and it does not have affinity for anions; the maltol-derivative **L25** forms a Gd^3+^ chelate of moderate stability and shows a moderate affinity for anions, depending on their relative basicity (phosphate > arsenate > bicarbonate > fluoride).

### 3.2. Tetra-Hydroxypyridinones

A group of researchers have lately devoted their work to the use of octadentate 1,2-HP compounds, in order to attain interesting potential environmental applications such as the separation of lanthanides from actinides, control of oxidation states and chelation capacity of 4f- and 5f- elements in reprocessing and storing of used nuclear fuel as well as chelating ability of other type of metal ions. Nuclear waste is generally composed in aqueous environment by actinides (e.g., U, Np, Pu, Am, Cm) and fission products (e.g., lanthanides, Cs, Tc). In aqueous medium, U mainly exists as the hexavalent linear uranyl cation, while Np and Pu predominate, respectively, in the pentavalent and tetravalent oxidation states. The complex composition of nuclear waste makes the respective treatment in the environment difficult, and so separative methods were proposed, corresponding to lab scale studies with certain mixtures of elements and not with nuclear waste, to avoid sequestration of varied metals by the proposed ligands, envisioning future potential industrial applications.

The understanding of the chelation ability and selectivity of these octadentate compounds is also important for their use as decorporation agents when internal exposure of working personnel to chemically and radiotoxic lanthanides/actinides occurs. Moreover, radiological contamination incidents may result in prevalent exposure to radiation in local and distant regions (e.g., 2011 Fukushima Daiichi Nuclear Plant accident).

**Figure 8:**
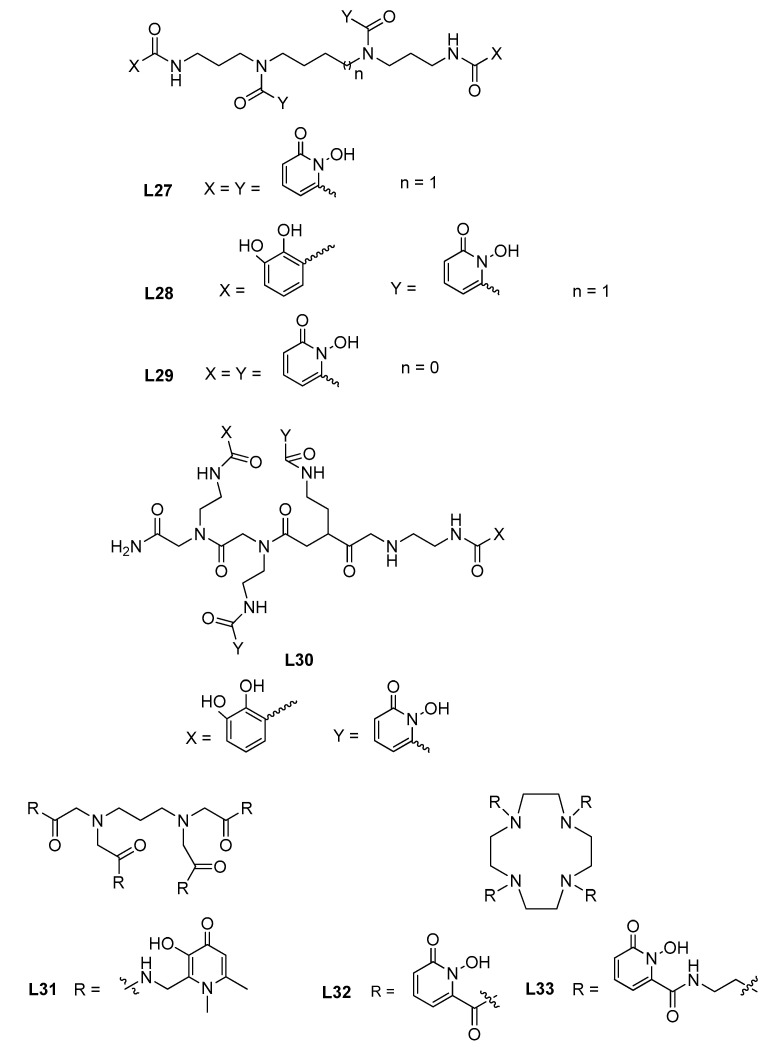
Molecular structure of selected octadentate compounds.

Among the tetra-1,2-HP compounds, the siderophore mimetic 3,4,3-LI-(1,2-HOPO) (**L27**, see Figure 8) appeared undoubtedly the most interesting recently studied chelator for actinides. **L27** was found to be able to promote oxo group activation via gas-phase chelation with the reduction in hexavalent NpO_2_^2+^ and PuO_2_^2+^ to tetravalent bis-hydroxo complexes. In this way, new actinyl complexes can be obtained by gas-phase two-electron actinyl reduction, otherwise not obtainable due to synthetic limitations [70]. The reduction in the NpO_2_^2+^/Np^4+^ redox pair (from +6 to +4), upon chelation with **L27** or with the bis-catecholamide analogue 3,4,3-LI(CAM)_2_(1,2-HOPO)_2_ (**L28**), was observed to be nearly instantaneous by X-ray absorption near edge structure (XANES) and extended X-ray absorption fine structure (EXAFS) [71]. This finding is important in terms of potential implementation of reprocessing of high-level nuclear waste (with considerable amount of long term radiotoxic ^237^Np), because the electron transfer kinetics of Np-oxo bond cleavage is known to be extremely slow both from the synthetical and environmental point of view. Although the usual destiny for Np is to be routed to high-level liquid waste (HLLW) for vitrification and further deposition in repositories, its recovery from HLLW has become interesting to both reduce the long term radiotoxicity of nuclear wastes as well as to obtain a heat source for radioisotope thermoelectric generators (^238^Pu). Therefore, an adapted PUREX (Plutonium Uranium Redox EXtraction) process was proposed, at the lab scale and with controlled solution composition, by using **L27**, which allows enhanced separation of Np from U and of Pu from U, with a separation factor (SF) of ca 7000 for both, therefore showing better performance, ca 90 and 10300 times higher, respectively, than the conservative PUREX process [72]. This results from the ability of **L27** to form a highly stable complex with Np as well as from its capacity to control the oxidation state of the metal ion by reducing it to Np^4+^. The long-lived fission products contained in HLLW (e.g., PUREX raffinate), such as lanthanides and some minor actinides (Np, Am, Cm), must be treated, in the reprocessing of used nuclear fuel, by adopting different methodologies and so it is of paramount importance to find methods able to separate these classes of radionuclides. The so-called *Trivalent Actinide Lanthanide Separation with Phosphorous-reagent Extraction from Aqueous Komplexes* (TALSPEAK) method is the reference one for separation of lanthanides(III) from actinides(III). This process typically uses di-(2-ethylhexyl)phosphoric acid (HDEHP) as the extractant and diethylenetriaminepentaacetic acid (DTPA) as the actinide(III) hold-back reagent, but it has some limitations such as narrow working pH range (3.5–4.0), expensive pH buffers and slow extraction kinetics. By using the in-lab model chelator **L27** with different industry-related organic extractants (e.g., Cyanex 301 GN, HDEHP), it was claimed that the TALSPEAK separation process of gadolinium from four trans-plutonium elements was ameliorated [73]: for HDEHP/**L27** combination (pH ca 1.5, lanthanides are extracted into organic phase and actinides are kept in aqueous phase), SF_Gd/Am_ = 30, SF_Gd/Cm_ = 8.5 and SF_Gd/Cf_ = 773; for Cyanex 301 GN/**L27** combination (optimal pH = 3.6, actinides are extracted into organic phase and lanthanides are kept in aqueous phase), SF_Am/Gd_ = 50 and SF_Cm/Gd_ = 23.

Since the significant mobility of uranyl (UO_2_^2+^) in environment can originate health risks derived from radiological contamination, complexation of U^4+^ and U^6+^ by **L27** was recently analyzed by UV-spectrophotometry, XANES and EXAFS. It was found that **L27**, as well as other spermine-derived analogues (e.g., **L28**), can stabilize U^4+,^ in aqueous media (inert complex towards oxidation/hydrolysis in acidic aqueous media) which is particularly relevant since tetravalent actinides are quite susceptible to hydrolysis even under very acidic conditions [74]. This study allows the further understanding of the conditions under which the uranium species are stable in solution, therefore contributing to preview the chemical behavior of radionuclides in diverse systems (chemical, biological or environmental). Compound **L28** also proved to be able to perform the double non-reductive activation of UO_2_^2+^, through Collision-Induced Dissociation (CID) fragmentation of the gas-phase complex, by cleaving off both strong U=O bonds and resulting in the formation of a formal U^6+^-**L28** chelate in the gas phase [75]. The obtained results allow us to wonder if **L28** and derivatives will be able to further implement actinyl-oxo activation through chelation in condensed phases.

**L27** is also being evaluated as a potential decorporation agent for actinides (first in human clinical phase). This compound turns out to be more potent than the only approved drug (DTPA) which has the disadvantage of being a non-specific ligand, with short retention time as well as low oral efficacy. Since internalized radionuclides (via inhalation or ingestion) rapidly attain the bloodstream, being primarily deposited in liver, bones and kidneys, chelation therapy must introduce a ligand in the body that can complex the harmful radionuclides (irrespective of their oxidation states), expel them in a complexed form and avoid the simultaneous removal of essential nutrient metal ions. In fact, the oxidation states of the actinides in vivo are uncertain upon complexation, and so in vitro structural and thermodynamic properties of complexes of **L27** with the actinide (III and IV oxidation states) series were recently studied (EXAFS and DFT) in order to obtain their corresponding structures, thermodynamic parameters, redox properties and electronic configurations [76]. **L27** is among the strongest actinide chelators and was found to be able, upon complexation, to both oxidize early actinide ions and reduce later ones in the series, a chemical behavior that can be important while designing future ligands able to separate actinides. The trans-plutonium actinide berkelium, formed in nuclear detonations or in nuclear fission reactors, is mainly present as Bk(III) in aqueous solutions and oxidizes to Bk(IV) under drastic conditions. **L27** was found to be able to stabilize the Bk(IV) complex even under very acidic conditions and since this neutral complex is not recognized by the human protein siderocalin, this could suggest new approaches to separate Bk from M(III) ions thus avoiding numerous steps and the use of strong oxidizers and harsh conditions (low pH, heating) [77]. In fact, good in-lab results were obtained for a proposed one-step separation method, for isolation of Bk from adjacent actinides and fission products by using HDEHP.HNO_3_-**L27** (SF_Bk/Lu_ = 320,000, and 3000–10,000 for Bk and adjacent Am^3+^, Cf^3+^ and Es^3+^) or TODGA.HNO_3_-**L27** (TODGA = *N*,*N*,*N*′,*N*′-tetraoctyldiglycolamide, SF_Cf/Bk_ > 1,000,000) systems, pointing towards the potential use of TODGA.HNO_3_-**L27** with high-activity samples and scaling up the production of high-purity Bk isotopes or to remove Bk traces in Cm, Cf or Es production [78]. Aiming at the purification of Ac from large amounts of Th, a DGA (diglycolamide) extraction chromatographic resin was employed in the presence of the selective tetravalent chelator **L27** in HNO_3_ medium, indicating that Th was fully complexed by **L27,** being unextracted [79]. Therefore, separation steps for trivalent actinides in the presence of significant amounts of Th can eventually be overcome by selective tetravalent complexation. An innovative colorimetric method, with eventual application in lanthanide detection in waste from industrial processes, was proposed based on the inhibition of gold nanoparticles growth by lanthanides in the presence of **L27** [80].

In order to further understand the coordination behavior of lanthanide versus actinide (III and IV oxidation states) metal ions with compounds **L27** and **L29**, diverse theoretical orbital-based and electron-density based methods were also employed, with the following inferences [81]: the HOPO core offers chelate-aromatic effects that gives stability to the chelates (Pu^4+^ > Th^4+^ > M^3+^, M ≡ trivalent metal ions), in full accordance with already reported experimental stability constants results [82,83]; the binding of lanthanides/actinides with the *hard* oxygen donor atoms of the ligands is of donor-acceptor type but a higher degree of covalency is found for actinides then for lanthanides resulting from the energy-driven orbital mixing between the actinide-5f orbitals with 2p orbitals of the oxygen donor atoms of the ligands.

Moreover, the widely studied compound **L27** continues to be evaluated as a chelating agent towards different metal ions and quite recently its binding interaction with rare earths (Sc^3+^ (log *β*_ScL_ = 25.16), Y^3+^ (log *β*_YL_ = 20.76)) [84], Hf^4+^ and Zr^4+^ (log *β*_ML_ > 42, complexes stable between pH 10 and 10 M HCl) [85], as well as a coordination study (X-ray absorption spectroscopy—XAS, luminescence measurements) with the heaviest element (einsteinium) that can be generated in quantities that enable classical macroscale studies have been performed [86]. Complexation studies of **L28** with Eu^3+^, Zr^4+^ and Th^4+^ (log *β*_EuL_ = 29.65, log *β*_ZrL_ = 57.26 and log *β*_ThL_ = 47.71) [87] evidenced the excellent chelating ability of this compound for both trivalent and tetravalent metal ions at the physiological pH. Moreover, the high affinities of these complexes by the human protein siderocalin (Scn) suggest a new radiopharmaceutical concept (Scn-**L28** system) for theranostic applications.

A new library of 16 octadentate peptoids based on hydroxypyridinone (1,2-HP) and cathecolamide sub-monomer units was developed for chelation of f-block metals (luminescence and thermodynamic solution studies with Eu^3+^ and Tb^3+^), by using a precision and systematic design approach with a solid-support synthesis [88]. This type of synthesis revealed to be a good investment for tailoring compounds towards efficient separation of *f*-elements (e.g., log *β*_EuL_ = 43.97 for **L30**). An octadentate 3,4-HP derivative compound (THPN, **L31**) demonstrated to have very high chelating capacities in aqueous solution towards zirconium and iron (pZr = 42.8 and pFe = 38) and to be a stronger Zr(IV) chelator than DFO (pZr = 32.2) although slightly weaker than **L27** (pZr = 44.0) [89]. Another innovative water-soluble rigid chelator for *f*-elements, a hydroxypyridinone(1,2-HP)-decorated macrocycle DOTHOPO (**L29**), showed rapid kinetics for the formation of stable complexes with lanthanides (Sm^3+^, Eu^3+^, Tb^3+^, Dy^3+^, pM = 19.9–20.8) and a late actinide (Cm^3+^, pCm = 21.9), and it can be used as a luminescence antenna for each metal ion [90]. In fact, this rigid cyclen backbone diminishes the movement of the pendant arms, therefore providing a closely packed coordination core for the octadentate Eu^3+^ metal ion of **L33**. This is believed to be responsible for its very high sensitization efficiency (64%), leading to less non-radioactive energy loss [91].

## 4. Polymeric and Dendrimeric Hydroxypyridinones

Recent achievements on polymeric and dendrimeric hydroxypyridinones have already been summarized in part in excellent reviews [3,5].

These macromolecular-hydroxypidinone chelators have been explored envisaging a broad range of potential medicinal and environmental applications, namely, to control ecotoxicity of *hard* metal ions as waste remediation.

Hydroxypyridinone bidentate ligands have been used as building blocks to construct polymeric matrices for the sequestering of *hard* metal ions, in particular, recently reported (3,4-HP)-functionalized sepharoses and silicas. The chelating sepharose gels, obtained via the reaction of *N*-alkylamine-3,4-HP’s and CNBr- or epoxy-activated sepharoses, showed high resistance against decomposition under highly acidic conditions highlighting their likely reusability as metal ion sorbents [92]. Studies on the metal complexing capabilities of these matrices revealed high affinity for a set of *hard* metal ions, with the following order affinity (Fe(III) > Th(IV) > Al(III)) in line with the determined stability constants (log *β*) of the MLH complexes with 3,4-HP (24.39, 23.22 and 20.87, respectively). Additionally, a new (3,4-HP)-functionalized silica, obtained through the reaction of *N*-aminopropyl-3,4-HP with mesoporous silica MCM41 and modified by reaction with (3-glycidyloxypropyl) trimethoxysilane showed excellent Fe(III)-binding capabilities. The covalently bound 3,4-HP containing novel material can be used as a sensor for trivalent iron [93].

**Figure 9:**
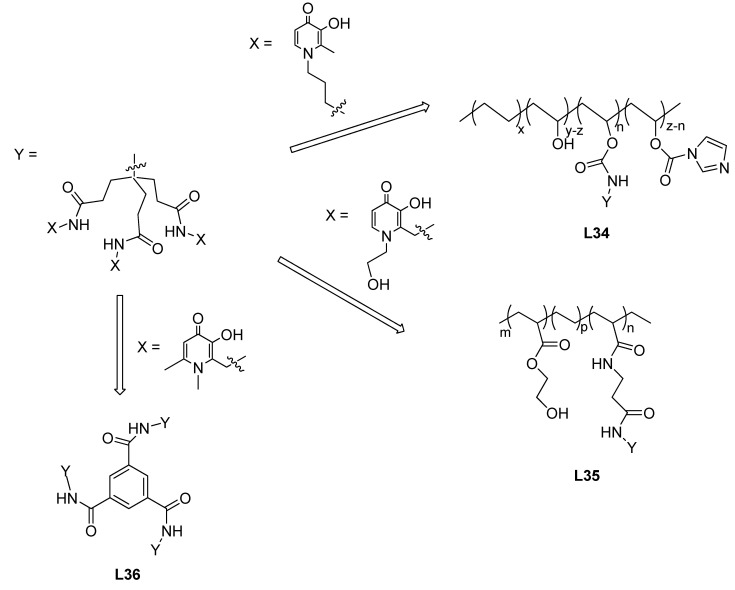
Schematic structure of (tris-3,4-HP)-containing copolymers (**L34**, **L35**) and dendrimer (**L36**).

In opposition to the bidentate ligand-containing polymers, very few reports are known about hexadentate chelator-containing polymers. However, these hexadentate-based polymers have expected advantages over the first ones, namely for the M^3+^ chelation, because they provide uniformly the necessary three binding units to complete the octahedral coordination. Therefore, some recent research has been focused on the development of hexadentate (3,4-HP)-containing polymers. In particular, aimed to obtain an analytical sensor for Fe(III), a copolymer was obtained from ethylene-vinyl alcohol and a tripodal, 3-hydroxy-4-pyridinone-based chelator (KC18) affording a thin film named 3,4-HP@EVOH (**L34,** Figure 9) [94]. Sorption isotherms demonstrated that the polymer is capable of sorbing Fe(III) even at low pH ~ 1. The moderate sorption capacities of the copolymer (order of 0.01 mmol/g) were explained by the complex formation between the active sites and Fe(III) occurring on the surface of the solid phase only. Based on desorption experiments, preliminary estimates of the formation constants of the 1:1 complexes with Fe(III) and the active sites of the copolymer strongly suggest the unaltered chelating properties of the ligands attached to the copolymer and the applicability of the obtained films as Fe(III) sensors.

With the hope of potential application for the treatment of wound infections a novel hexadentate 3,4-HP-based ligand incorporating a C=C bond too was synthesized and its copolymer with 2-hydroxyethyl acrylate HEA (**L35**, Figure 9) was also prepared and evaluated [95]. The monomeric ligand showed high iron(III) affinity with a stability constant of the FeL complex being log *K*_1_ = 33.61. Both the monomeric chelator and the polymer were found to possess antimicrobial activity against both Gram-positive and Gram-negative bacteria based on inhibition zone and minimum inhibitory concentration assays due to their very high Fe(III) sequestering capabilities.

The same group has also studied the iron-binding and antimicrobial properties of hexadentate 3,4-HP-terminated dendrimers. In this class of macromolecular chelators, dendrimers, in opposition to polymers, have a single molecular structure, thus being more reproducible under controlling quality parameters. The dendrimers can be synthesized using a divergent or a convergent approach, to afford the first and second generation of dendrimers, respectively. While the “first generation” dendrimers contained three tripodal, hexadentate 3,4-HP units **(L36**, Figure 9) the “second generation” dendrimers can have six or nine of them [5,96]. UV-Vis titration study (a competition method using a fluorescent chelator for the dendrimers) demonstrated that each hexadentate 3,4-HP-containing unit is capable of binding one Fe(III) in complexes with high stability both for the monomeric building block ligands and in the dendrimers. The calculated pFe values (in the range 29.8–30.3) indicated almost identical iron(III)-binding strengths for the chelating units. The in vitro inhibitory effect of the dendrimeric iron chelators on the growth of two Gram-negative bacteria (*E. coli* and *P. aeruginosa*) and two Gram-positive bacteria (*S. aureus* and *B. subtilis*) was evaluated. All the chelators were found to have marked inhibitory effect on the tested bacteria [96].

## 5. Miscellaneous Macromeric and Nanomeric HP-Based Chelators

Removal of uranium from sea water as a fuel supply for nuclear energy is an emerging area nowadays. For this purpose, using click reaction, *N*-alkylamido-3,4-HP was grafted to the surface of modified nanoporous silica. The obtained sorbent integrating high binding affinity and high surface area nanostructures showed excellent collection performance for uranium. Stripping of uranium from the sorbent was accomplished with the inexpensive and nontoxic sodium carbonate solution in the form of [UO_2_(CO_3_)_3_]^4^^−^ [97].

Actinides possess both chemotoxicity and radiotoxicity. Their radiotoxicity may cause direct radiation-induced organ damage and indirect damage, mostly through radiation-induced reactive oxygen species (ROS). To address both of these challenges, 3,2-HP-grafted chitosan oligosaccharide nanoparticles (NPs) have been synthesized and evaluated as efficient decorporation agents capable of removing both uranium(VI) and ROS in vivo. Based on cellular cytotoxicity and animal decorporation assays it was demonstrated that the novel NPs exhibit a remarkable decrease in toxicity and promotion of the uranium removal capability from both kidneys and femurs. The decorporation efficacy could reach up to 43% in epithelial cells (NRK-52E), 44% in kidneys and 32% in femurs. The ROS levels of the cells treated with NPs were also found significantly lower than those of the control group, implying a promising radiation protection effect [98].

A potent 1,2-HP-based octadentate chelator was encapsulated in chitosan NPs in order to be used as an actinide decorporation agent via inhalation. Compared to the unformulated chelator, encapsulating with biocompatible, biodegradable nanoparticles made of chitosan resulted in an extended-release profile of the chelating agent to lung fluid in in vitro experiments. This could lead to a reduction in the dosing frequency required to achieve the decorporation efficacy of unformulated chelator ligand itself [99].

For the selective and almost instant monitoring of uranyl ([UO_2_(H_2_O)_4_]^2+^) ions a 3,4-HP-functionalized carbon quantum dot was synthesized and tested. The developed fluorescent probe exhibited an excellent sensitivity (LOD = 6.53 ppb), a high selectivity towards uranyl ions and an extremely short response time (30 s) [100].

A new library of sixteen, tetrameric, octadentate peptoids based on 1,2-HP- and catecholamide-based sub-monomers, and for comparison, the corresponding mixed ligands derived from the spermine scaffold were prepared and studied for the chelation of f-block metals. Coordination-based luminescence studies were carried out with Eu^3+^ and Tb^3+^ and revealed higher sensitization efficiency with the spermine scaffold. Both ligand systems showed high affinity for Ln(III) metals with stability constants greater than 10^29^ for Eu^3+^ [88].

Tripodal, hexadentate 3,4-HP-based ligands capable of binding ^68^Ga(III) and multiple, αvβ3 integrin-targeted c(RGDfK) peptide groups were incorporated into a single molecule, thus increasing the affinity of the radiolabeled conjugate for receptors in vivo as well as increasing the specific activity of the radiolabeled species itself. In contrast to previous work on multimeric RGD peptide conjugates, the new dendritic system possesses tris(hydroxypyridinone) chelators that coordinate ^68^Ga(III) quantitatively at ambient temperature, near neutral pH and micromolar concentrations of conjugate, allowing for rapid, one-step labeling [101].

The synthesis of a novel water-soluble low molecular mass iron sequestering co-polymer (DIBI) containing 3,4-HP methacrylamide monomer and *N*-vinylpyrrolidone was reported. The polymer displays 100 to 1000 times lower MICs in vitro against the bacteria *S. aureus* and *A. baumannii*, and also the fungus *C. albicans* compared to the clinically used iron chelators, DFO and DFP. The authors hypothesized that the enhanced antimicrobial activity of DIBI is due to the inability of microbes to internalize the polymer and to the intramolecular proximity of neighboring five binding moieties [102].

The recently developed iron-binding polymer, DIBI, was successfully applied in animal experiments for the treatment of non-infectious uveitis, an inflammatory disease of the eye. Since a main mediator of inflammation are oxygen free radicals generated in iron-dependent pathways, DIPI with excellent Fe(III)-binding capabilities exerted an anti-inflammatory effect in local and systemic models of endotoxin-induced uveitis [103].

DIBI was also shown to inhibit the growth of five different breast cancer cell lines (SK-BR3, MDA-MB-468, MDA-MB-231, MCF-7, and T47D). Detailed screening of the MDA-MB-468 cells revealed that iron withdrawal was associated with increased expression of transferrin receptor 1 and ferritin H mRNA but decreased expression of ferroportin mRNA. Combination treatment of MDA-MB-468 cells with DIBI and cisplatin caused greater DNA damage than treatment alone and suggests that DIBI-mediated iron withdrawal may enhance the effect of chemotherapeutic agents used in breast cancer treatment [104].

## 6. Concluding Remarks and Perspectives

Since about five decades ago, but in particular along the last two decades after the approval of a hydroxypyridinone (HP) chelating drug (DFP) for the treatment of iron overload situations, intensive research has been focused on the broad family of HP-based chelators for *hard* metal ions and their stable metal complexes, aiming to achieve a great diversity in medical and environmental applications. Although a major recent interest has been focused on ligand structural modifications to make them better suited for the pharmacological requirements of therapeutic or diagnostic applications, the development of HP-based chelators for environmental applications is also receiving an increasing considerable attention. Their potential use envisages the remediation of *hard* metal ion ecotoxicity in living beings or other biological systems characterized by accumulation of toxic metal ions due to environmental stresses such as nuclear power waste. The analysis of recent literature reports (last 5–6 years) shows that diverse structural modifications have been used (schematically presented in Figure 1). Originally, simple bidentate mono-HPs were used but quite often extra-functionalized to tune the necessary lipo-hydrophilic balance or to aid the targeting of specific biosites. Meanwhile, several strategies for the design of multi-HP-based chelators have been followed by appending two, three or multiple HP moieties to a diversity of soluble or insoluble anchoring skeletons (polymers and dendrimers) therefore increasing the metal chelating efficacy to optimize the thermodynamic/kinetic stability of the metal complexes and to reduce ligand dosage and related toxicity. The huge number of new discoveries in this area, recently reported in the literature, illustrates the tremendous broad potential of hydroxypyridinone-based chelators. In fact, besides their ability to form very stable complexes with *hard* metal ions, their easy preparation and functionalization make them “privileged” chemical tools to be included as building blocks in the design of strong chelating systems. Future developments seem to point towards the further design and development of the interesting multi-HPs with maximization of the chelation power, although it may still involve solubility issues for some envisaged applications. Therefore, to proceed with this, challenging research appears to have extreme relevance to support the environmental/medical sciences in the control and remediation of severe situations of *hard* metal ecotoxicity associated with the increasing amount of serious environmental insults and disasters worldwide that may eventually result in prevalent exposure to radiation in local or even distant regions.

## Figures and Tables

**Table 1:** pM values reported in literature for the mono-3-hydroxy-4-pyridinones.

Mono-3,4-HP	pAl [7]	pFe [8]	pCu [8]	pZn [10]	pCa [9]	pMg [9]
**L1**	14.7	-	8.6	6.4	6.0	-
**L2**	14.2	24.4	11.0	6.0	6.0	6.0
**L3**	13.2	-	11.1	6.0	6.0	-
**L4**	13.9	-	10.9	6.0	6.0	6.0
**L5**	13.2	24.0	8.2	6.2	6.0	6.0

**Table 2:** LOD and LOQ values determined using hydroxypyridinone ligands for Fe^3+^ assay.

HP Ligand	Analytical Method	LOD/μM	LOQ/μM	Ref.
**L6**	Colorimetry	3.58	12.53	[25]
**L7a**	µSI-LOV ^a^ system	0.32	1.07	[18]
**L7b**–**L7e**	Sequential injection	1.24–1.79	-	[27]
**L7a**	μPAD ^b^ system	0.98	4.47–35.81	[28]
**L7b**	Spectrophotometry with on-line SPE ^c^, NTA resin column	0.05	-	[29]
**L7b**	Spectrophotometry without on-line SPE ^c^	0.20	-	[29]

^a^ µSI-LOV: microsequential injection lab-on-valve; ^b^ μPAD: microfluidic paper-based analytical device; ^c^ SPE: solid-phase extraction.

**Table 3:** pM values reported in literature for bis-3,4-HPs and commercial metal chelators.

Compounds	pFe	pAl	pZn	Ref.
**L10**	26.6	21.4	17.4	[45]
**L11**	20.6	18.2	13.9	[46]
**L12**	25.8	19.1	8.3	[47]
DFP **(L8b)**	19.3	16.1	6.2	[14]
DFO	26.5	19.3	6.5	[48]
Transferrin	20.3	14.5	-	[49,50]
NTA	18.5	12.8	-	[51]
EDDA	17.1	13.1	11.1	[51]
DTPA	24.6	15.2	14.8	[51]
DOTA	24.3	13.2	-	[51]
IDAPr(3,4-HP)_2_	25.8	18.8	9.7	[52]
EDTAPr(3,4-HP)_2_	26.5	19.0	10.7	[53]
IDAPipPr(3,4-HP)_2_	25.7	18.0	-	[54]
IDAPipPr(3,4-HP)_2_ + **L4**	26.5	17.9	-	[54]

## Data Availability

Data reviewed in this work were taken from the cited references.
